# Chemical Composition, Antioxidant and Enzyme Inhibitory Activities of *Onosma bourgaei* and *Onosma trachytricha* and in Silico Molecular Docking Analysis of Dominant Compounds

**DOI:** 10.3390/molecules26102981

**Published:** 2021-05-18

**Authors:** Erman Salih Istifli

**Affiliations:** Department of Biology, Faculty of Science and Literature, Cukurova University, TR-01330 Adana, Turkey; esistifli@cu.edu.tr; Tel.: +90-537-437-05-67

**Keywords:** *Onosma bourgaei*, *Onosma trachytricha*, LC-MS/MS, antioxidant, enzyme inhibition, molecular docking

## Abstract

The aim of this study was to investigate the chemical composition, antioxidant and enzyme inhibitory activities of methanol (MeOH) extracts from *Onosma bourgaei* (Boiss.) and *O. trachytricha* (Boiss.). In addition, the interactions between phytochemicals found in extracts in high amounts and the target enzymes in question were revealed at the molecular scale by performing in silico molecular docking simulations. While the total amount of flavonoid compounds was higher in *O. bourgaei*, *O. trachytricha* was richer in phenolics. Chromatographic analysis showed that the major compounds of the extracts were luteolin 7-glucoside, apigenin 7-glucoside and rosmarinic acid. With the exception of the ferrous ion chelating assay, *O. trachytricha* exhibited higher antioxidant activity than *O. bourgaei*. *O. bourgaei* exhibited also slightly higher activity on digestive enzymes. The inhibitory activities of the *Onosma* species on tyrosinase were almost equal. In addition, the inhibitory activities of the extracts on acetylcholinesterase (AChE) were stronger than the activity on butyrylcholinesterase (BChE). Molecular docking simulations revealed that luteolin 7-glucoside and apigenin 7-glucoside have particularly strong binding affinities against ChEs, tyrosinase, α-amylase and α-glucosidase when compared with co-crystallized inhibitors. Therefore, it was concluded that the compounds in question could act as effective inhibitors on cholinesterases, tyrosinase and digestive enzymes.

## 1. Introduction

The members of the Boraginaceae are mainly spread in tropical regions of the world. According to the taxonomic records, this genus is estimated to consist of 100 genera and 2000 species spread throughout the Earth [1,2]. Many *Onosma* species have been scrutinized to determine their biologically active substances such as deoxyshikonin, acetyl shikonin, 3-hydroxyisovaleryl shikonin and 5,8-O-dimethylacetyl shikonin [2]. Shikonin derivatives, known to be produced by some members of *Onosma*, are used as natural dyes due to their red color and have also been used in the silk and food industries since ancient times [3,4,5]. The cultivation of *Onosma* species, therefore, is of great importance. Additionally, many *Onosma* species have been found to have several activities such as antioxidant, antimicrobial, wound healing [6,7], and anti-tumor [7]. On the other hand, some *Onosma* species (*O. argentatum*, *O. echiodes*, *O. heterophylla*, and *O. stellulatum*) have been reported to contain alkaloids showing carcinogenic and/or hepatotoxic properties [8,9].

Antioxidant activity is one of the most important pharmacological properties of plants and plants are rich sources of antioxidant phytochemicals. Many researchers agree that oxidative stress is strongly correlated with the pathology of many diseases [10]. Antioxidants provide significant advantages in dealing with the negative effects of oxidative stress on the body. Polyphenols are phytochemicals that stand out with their antioxidant activities and are abundant in many vegetables and fruits that we consume in our daily lives [10]. Scientific studies have shown that consuming foods rich in polyphenols reduces the risk of many common diseases such as degenerative disorders, diabetes, cardiovascular diseases, and cancer [10,11,12,13]. Polyphenols not only protect the body from oxidative stress, but also known to exhibit significant inhibitory activity on α-glucosidase and α-amylase, the enzymes responsible for carbohydrate digestion. Inhibition of these enzymes is considered to be an important approach in medical treatment as they suppress postprandial hyperglycemia in diabetic patients [14]. Therefore, it is thought that the determination of new plant species and bioactive compounds that have an inhibitory effect on these enzymes will lead to promising advances in the treatment of diabetes [15].

In addition to the inhibition of digestive enzymes mentioned above, plants also have numerous phytochemicals showing tyrosinase inhibitory activity, which has become increasingly popular in recent years. Melanin is an important barrier that protects our body against harmful rays [16]. The synthesis of this pigment is catalyzed by tyrosinase under the stimulation of UV rays [17,18]. Excessive melanin synthesis leads to hyperpigmentation-related disorders. [19]. Excessive melanin synthesis can be prevented through the use of tyrosinase inhibitors. Tyrosinase inhibitors are one of the newest approaches used in the cosmetic industry today to whiten the skin. It is known that kojic acid, mercury, hydroquinone, and arbutin, whose tyrosinase inhibitor activities are well-characterized, have serious side effects on our body in long-term use. For this reason, both the leading companies of the cosmetics industry and researchers have focused on discovering new and more effective phytochemicals that do not cause serious adverse effects in the human body [20].

Acetylcholine (ACh) is an important neurotransmitter responsible for the regulation of normal memory functions [21]. However, the amount of this substance can decrease dramatically, especially in the elderly individuals, due to the hydrolysis by cholinesterases (ChEs), which are responsible for the regulation of the amount of ACh [22]. Therefore, inhibition of ChEs is a useful strategy in these individuals in order to prevent the decrease in the amount of neurotransmitters and to stabilize cognitive functions [5,23,24,25,26,27,28].

The purpose of this study was to determine the chemical composition of methanol (MeOH) extracts obtained from *O. bourgaei* (Boiss.) and *O. trachytricha* (Boiss.) and to compare their antioxidant and enzyme inhibitor activities on α-amylase, α-glucosidase, tyrosinase and ChEs. In addition, to provide further insights into enzyme inhibitory activity, interactions between dominant compounds and enymes in question were studied via molecular docking.

## 2. Results

### 2.1. Antioxidant Activity of the Extracts

**Figure 1:**
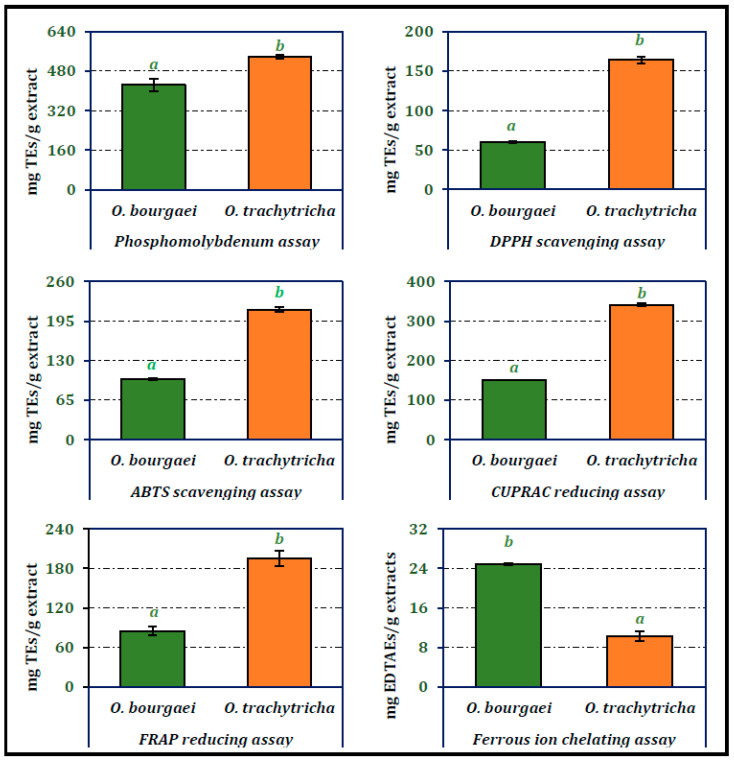
Total antioxidant, radical scavenging, reducing power and chelating activity of the MeOH extracts of *O. bourgaei* and *O. trachytricha* [TEs: Trolox equivalents, EDTAEs: Ethylenediaminetetraacetic acid (disodium salt)]. Data with different superscripts (a, b) were different from each other. All tests were performed in triplicate.

The antioxidant activities of the MeOH extracts of *Onosma* species were analyzed using phosphomolybdenum, DPPH and ABTS radical scavenging, CUPRAC and FRAP reducing power potential and ferrous ion chelating assay techniques (Figure 1). *O. trachytricha* exhibited higher antioxidant activity than *O. bourgaei* in all parameters except ferrous ion chelating assay. Phosphomolybdenum, DPPH and ABTS scavenging and CUPRAC and FRAP reducing potentials of *O. trachytricha* were determined as 536.69, 163.92, 213.88, 340.78, and 195.50 mg TEs/g extract, respectively. However, in chelating activity assay, the activity of *O. bourgaei* (24.85 mg EDTAEs/g extract) was higher than that of *O. trachytricha* (10.33 mg EDTAEs/g extract). The higher activity of *O. trachytricha* which was richer in the selected phytochemicals, in most of the antioxidant activity tests showed that the chemical composition contributed to the antioxidant activity significantly. In all of the antioxidant activity tests, both extracts exhibited different activities that are statistically significant.

### 2.2. Enzyme Inhibitory Activity of the Extracts

**Figure 2:**
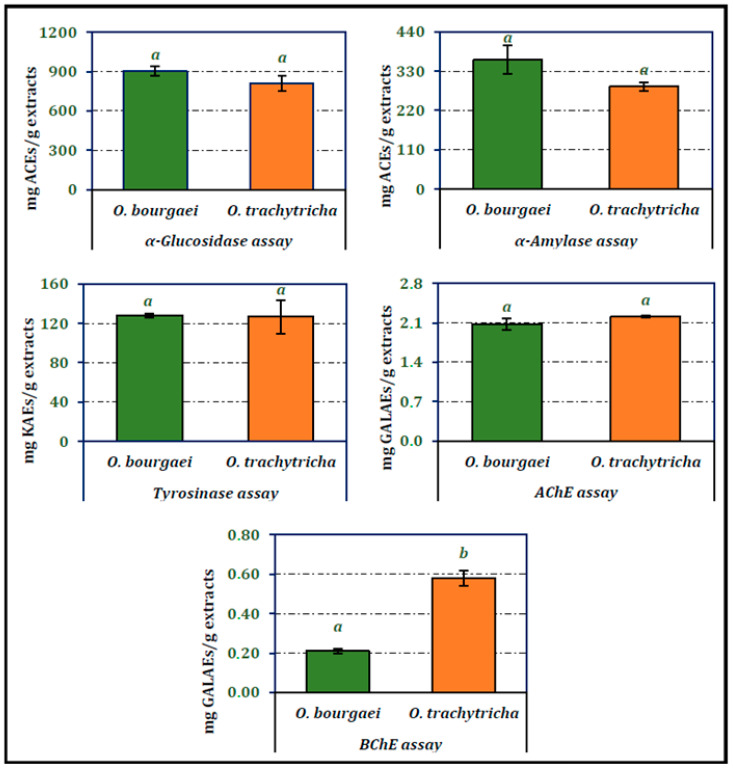
Enzyme inhibition activities of the MeOH extracts of *O. bourgaei* and *O. trachytricha* (KAEs: Kojic acid equivalents, GALAEs: Galanthamine equivalents, ACEs: Acarbose equivalents). Data with different superscripts (a, b) were different from each other. All tests were performed in triplicate.

Inhibitory activities of the MeOH extracts obtained from *O. bourgaei* and *O. trachytricha* on α-glucosidase, α-amylase, tyrosinase, AChE and BChE are presented in Figure 2. *O. bourgaei* exhibited higher inhibitory activity on digestive enzymes than other *Onosma* species (902.99 and 361.64 mg ACEs/g extract, respectively). The activity of the extracts on tyrosinase was almost equal. *O. trachytricha* exhibited higher inhibitory activity on both cholinesterases than *O. bourgaei* (2.21 and 0.58 mg GALAEs/g extract, respectively). Furthermore, in BChE inhibitor activity assay, the inhibitory activity of *O. trachytricha* was more than two folds higher than that of *O. bourgaei*.

### 2.3. Phytochemistry of O. bourgaei and O. trachytricha

**Figure 3:**
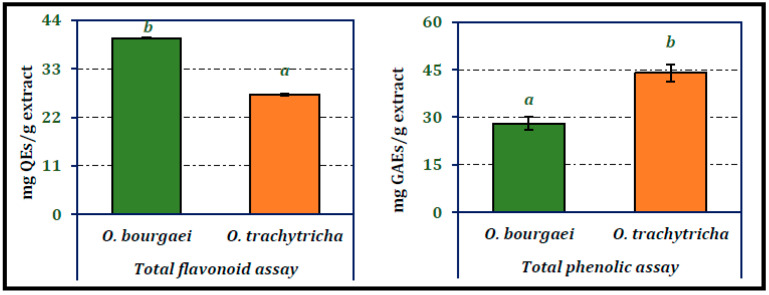
Total phenolic and flavonoid contents of the MeOH extracts of *O. bourgaei* and *O. trachytricha* (QEs: Quercetin equivalents, GAEs: Gallic acid equivalents). Data with different superscripts (a, b) were different from each other. All tests were performed in triplicate.

Chemical composition of *Onosma* species were analyzed using both qualitative and quantitative techniques. Total phenolic and flavonoid compounds of *Onosma* species were analyzed spectrophotometrically. According to data presented in Figure 3, *O. bourgaei* was richer in flavonoids, while *O. trachytricha* was found to contain higher amount of phenolics. The amounts of flavonoids in the extracts were 39.92 and 29.09 mg QEs/g extract, respectively. On the other hand, phenolic amounts of MeOH extracts were determined as 28.05 and 43.97 mg GAEs/g extract, respectively. The phenolic and flavonoid contents of each extract were statistically significantly different from each other.

**Figure 4:**
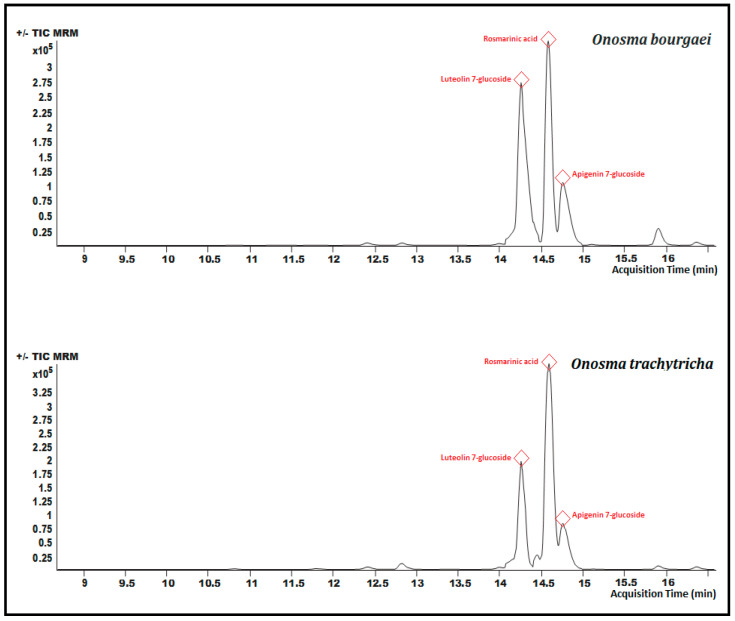
Chromatograms of the MeOH extracts of *O. bourgaei* and *O. trachytricha.* The three major components were depicted in red color.

Chromatographic analyzes were carried out to determine the amounts of some phytochemicals in the extracts. The analytical characteristics of these phytochemicals are presented in Table 1. The LC-MS/MS chromatograms of selected phytochemicals in MeOH extracts were given in Figure 4 and as µg/g extract in Table 2. As can be seen from the chromatograms in Figure 4, the main compounds of both *O. bourgaei* and *O. trachytricha* were luteolin 7-glucoside (23,908.22 and 22,326.01 µg/g extract, respectively), rosmarinic acid (10,013.76 and 24,837.51 µg/g extract, respectively) and apigenin 7-glucoside (21,689.17 and 17,949.03 µg/g extract, respectively). Many compounds selected were in high quantities in *O. trachytricha* extract. However, it was found that some phytochemicals such as pinoresinol, luteolin 7-glucoside, gallic acid, luteolin, eriodictyol, apigenin 7-glucoside, and verbascoside were in higher amounts in *O. bourgaei* extract than *O. trachytricha.*

### 2.4. Molecular Docking Studies

**Figure 5:**
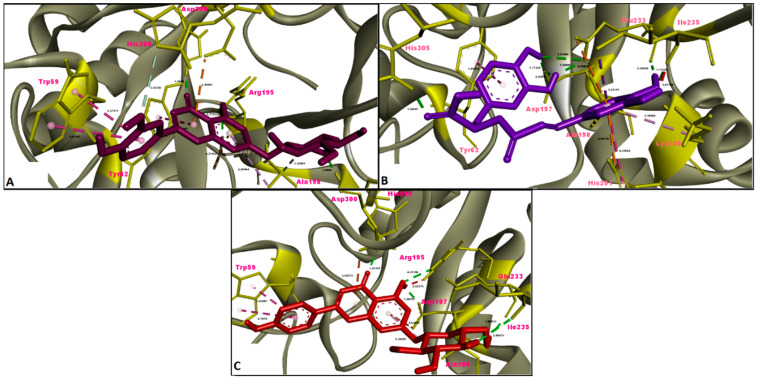
Molecular interaction between acetylcholinesterase (AChE) and (**A**) luteolin 7-glucoside, (**B**) rosmarinic acid and (**C**) apigenin 7-glucoside.

**Figure 6:**
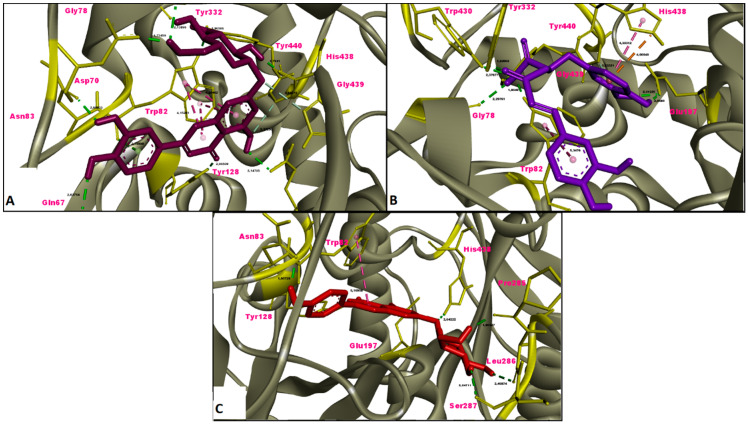
Molecular interaction between butyrylcholinesterase (BChE) and (**A**) luteolin 7-glucoside, (**B**) rosmarinic acid and (**C**) apigenin 7-glucoside.

**Figure 7:**
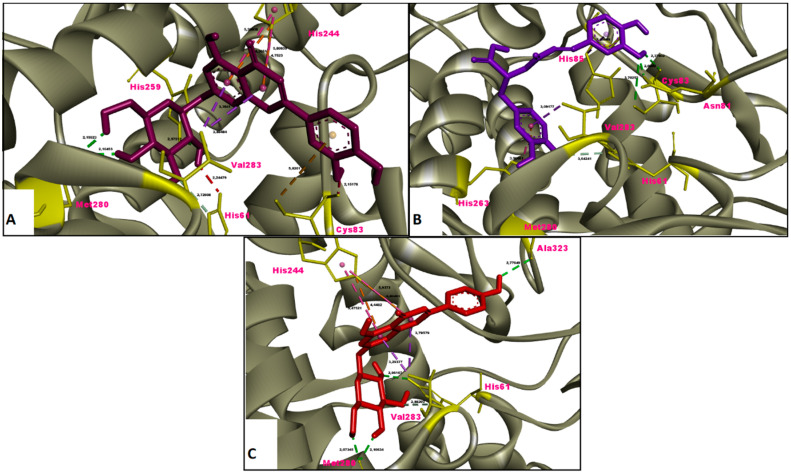
Molecular interaction between tyrosinase and (**A**) luteolin 7-glucoside, (**B**) rosmarinic acid and (**C**) apigenin 7-glucoside.

**Figure 8:**
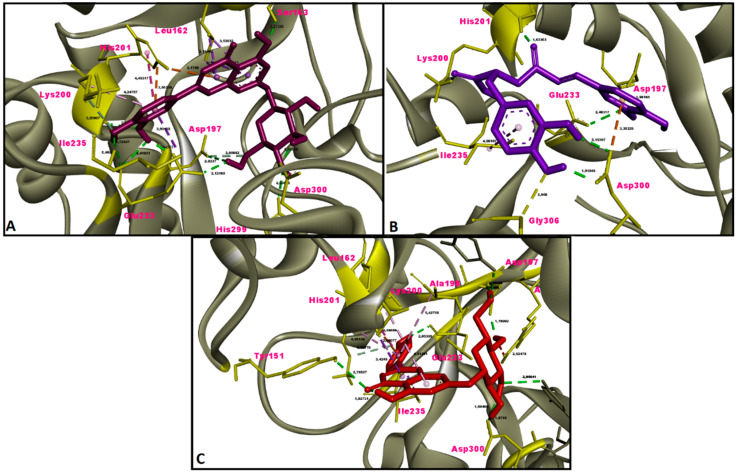
Molecular interaction between α-amylase and (**A**) luteolin 7-glucoside, (**B**) rosmarinic acid and (**C**) apigenin 7-glucoside.

**Figure 9:**
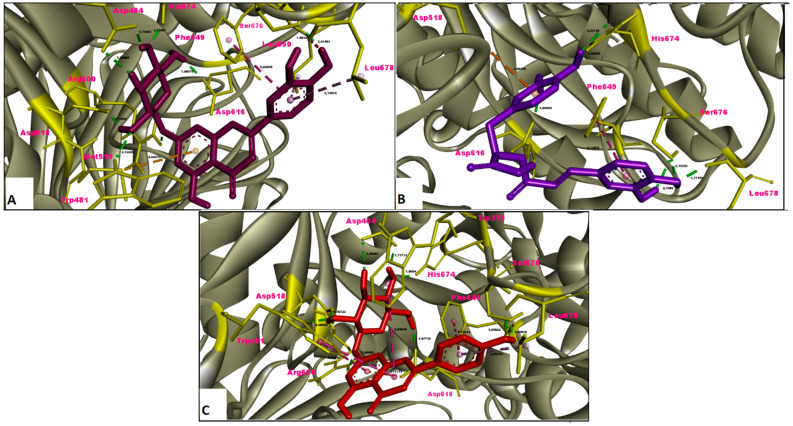
Molecular interaction between α-glucosidase and (**A**) luteolin 7-glucoside, (**B**) rosmarinic acid and (**C**) apigenin 7-glucoside.

In this study, the results of molecular docking analysis of the three major phytochemicals with their binding affinities (kcal/mol) and inhibition constants (mM) against AChE, BChE, tyrosinase, α-amylase and α-glucosidase were given in Table 3. In addition, the binding affinity values of the inhibitors obtained by performing redocking of the co-crystallized ligands of these enzymes were used as positive control values for comparison. Luteolin 7-glucoside and apigenin 7-glucoside generally exhibited high binding affinities for these five enzymes. The binding affinities and inhibition constants of these two flavonoid glycosides were found to be comparable to or even more negative than the positive control of each enzyme (Table 3). Rosmarinic acid, on the other hand, displayed the most remarkable binding affinity for BChE enzyme (ΔG° = −7.91 kcal/mol), however, it did not show strong binding affinity as the positive control for the other four enzymes. Flavonoid glycosides (luteolin 7-glucoside and apigenin 7-glucoside), on the other hand, specifically exhibited noticeable binding affinity on the AChE, BChE, tyrosinase and digestive enzymes and these binding affinities were comparable to or even more negative than the positive controls (Table 3). The aminoacid residues in which the ligands interact within the inhibitor binding pockets of the enzymes AChE, BChE, tyrosinase, α-amylase and α-glucosidase are shown in Figure 5, Figure 6, Figure 7, Figure 8 and Figure 9. The results obtained from molecular docking calculations show that luteolin 7-glucoside and apigenin 7-glucoside have particularly strong binding affinities when compared with co-crystallized inhibitors (galantamine, tacrine, kojic acid and acarbose) (Table 3).

## 3. Discussion

As far as could be ascertained from a literature survey, the chemical composition, antioxidant and enzyme inhibitory activities of the *Onosma* species evaluated here have not previously been reported elsewhere. Therefore, data presented here could be assumed as the first report on phytochemicals and aforementioned activities of these species. However, there are some literature data regarding the contribution of phytochemicals, which were determined as the major compounds in the current study, to antioxidant and enzyme inhibitory activity. These literature data were discussed in detail below.

In previous studies, it was reported that some *Onosma* and *Anthemis* species were to be rich in apigenin 7-glucoside showing significant antioxidant activity [5,26,29,30]. There were also some studies in the literature published by other research groups that this compound may positively affect antioxidant activity. Ozcan et al. [31] have investigated the effect of fermentation and bud size on chemical composition and antioxidant activity in capers. They found that the antioxidant activity of fresh material was higher than that of fermented one and apigenin 7-glucoside was reported to be one of the main compounds of the fresh material. In another study investigating the antioxidant and protective effects of *Rhanterium suaveolens* on mouse erythrocytes against acetamiprid (ACT)-induced oxidative stress, significant increases in SOD, CAT, and GPx activities were found in mice treated with *R. suaveolens* at a dose of 300 mg/kg. HPLC-DAD analysis showed that one of the main compounds of the extract was apigenin 7-glucoside and this compound can affected the activity positively [32].

As with apigenin 7-glucoside, previous studies also revealed that various *Onosma* species were also rich in luteolin 7-glucoside exhibiting significant antioxidant activities [5,26,29,30]. Additionally, it has been reported that the free radical scavenging and ferrous ion chelating activities of the hydro-alcoholic extract obtained from *Fraxinus angustifolia* were quite successful compared to vitamin E, even acting as an effective chain-breaking antioxidant against lipoperoxyl radicals. In that study, HPLC-DAD analyzes showed that luteolin 3,7-glucoside was among the major compounds [33].

Essafi et al. [34] have attempted to correlate the activities and chemical compositions of Tunisian olive leaf extracts. They reported that the main components were luteolin 4-glucoside, luteolin 7-glucoside and apigenin 7-glucoside and these compounds exhibited significant DPPH radical scavenging activity. The antioxidant activity of rosmarinic acid has been reported by many researchers [35,36,37,38,39]. Therefore, rosmarinic acid was considered to have a positive effect on antioxidant activity. The literature data discussed above corroborate those derived from the present study.

There are various reports in the literature that rosmarinic acid and flavonoid glycosides showed inhibitory effect on digestive enzymes. Witkowska-Banaszczak et al. [40] reported that *Succisa pratensis* is an important herbal source containing luteolin and apigenin 7-glycosides, and these flavonoid glycosides isolated from the flowers and leaves of this plant significantly inhibit the activity of pancreatic α-amylase. Findings from the study conducted by Ma et al. [41] also supported those reported by Witkowska-Banaszczak et al. [40]. In the study reported by Ma et al. [41], as a result of the chemical characterization of the polyphenols of *Sphallerocarpus gracilis* stems and leaves, luteolin 7-glucoside was identified as the main component, and the extract was reported to have a dose-dependent inhibitory effect on α-glucosidase. According to the literature data, rosmarinic acid is also among the phytochemicals that show inhibitory activity on digestive enzymes. Deng et al. [42] reported that *Ehretia macrophylla*, which is rich in rosmarinic acid, showed promising hypoglycemic activity by inhibiting α-glucosidase and α-amylase.

The tyrosinase inhibitory activities of flavonoid glycosides, which were reported as the major compounds in the present study, have not been studied before. However, in previous studies, some plant species rich in these flavonoid glycosides have been found to exhibit significant tyrosinase inhibitory activity [5,26,29,30,43]. However, data reported by Bouzaiene et al. [44] was in contradiction with the literature data presented above. According to Bouzaiene et al. [44], apigenin 7-glucoside increased tyrosinase activity in B16F10 melanoma cells and, consequently, melanin synthesis. To better understand the inhibitory activities of the flavonoid glycosides in question on tyrosinase, it was thought that the individual activities of these compounds should be tested. In contrast to the flavonoid glycosides mentioned above, there was evidence that rosmarinic acid has tyrosinase inhibitory activity. Monophenolase and diphenolase inhibitory activities of rosmarinic acid isolated from ethanol (EtOH) extract of *Lepechinia meyenii* were reported to be 4.14 and 8.59 µM, respectively [45]. However, in molecular docking calculations, a strong binding affinity of rosmarinic acid with tyrosinase was not found.

The cholinesterase inhibitory activities of flavonoid glycosides have not been studied before. Therefore, as stated above, further testing of individual inhibitory activities of these phytochemicals will help to elucidate the chemical composition-activity relationship in future studies. However, published data on the inhibitory activities of rosmarinic acid are clearer. Bilska et al. [46] have studied the activities of *Rosmarinus officinalis* of which major compound was rosmarinic acid. They reported that the AChE inhibitory activity of rosemary was higher than BChE inhibitory activity. This difference in inhibitory activity against cholinesterases was in correlation with those obtained from the present study.

In the present study, molecular docking calculations were also performed to evaluate the inhibitory activity of the main components on the enzymes in question. In the comparison of inhibitory activity, binding affinity values of co-crystallized ligands of these enzymes were used as positive controls. Molecular docking is a key technique in structural molecular biology and its purpose is to predict the primary (predominant) binding mode(s) and binding affinity of a ligand that complexes with a protein of known three-dimensional structure [47]. In this study, molecular docking analyzes were performed to estimate the binding modes and binding affinities of the identified three major compounds with tyrosinase, α-amylase, α-glucosidase and ChEs enzymes. The binding affinity (ΔG°; kcal/mol) and inhibition constant (K_i_, mM) values of main compounds against studied enzymes were shown in Table 3 and the top ranked receptor-ligand conformations were given in Figure 5, Figure 6, Figure 7, Figure 8 and Figure 9. Of particular importance, the flavonoid glycosides (luteolin 7-glucoside and apigenin 7-glucoside) showed highly favourable free energy of binding against ChEs, tyrosinase, α-amylase and α-glucosidase when compared with positive controls. Thus, the data from this study show that the flavonoid glycosides can act as effective inhibitors on cholinesterases, tyrosinase and digestive enzymes.

## 4. Materials and Methods

### 4.1. Plant Material

The aerial parts of *Onosma bourgaei* Boiss. (1608 m., 40°33′50″ N 39°24′15″ E, Herbarium number: OC.5045) and *Onosma trachytricha* Boiss. (1169 m., 40°31′48″ N 39°23′39″ E, Herbarium number: OC.5046) were collected from Gumushane-Turkey in 2019. The plants were identified and deposited by Dr. Olcay Ceylan from the Department of Biology, Mugla Sitki Kocman University, Mugla-Turkey.

### 4.2. Extraction Process

Samples of five grams from the dried aerial parts were weighed and macerated in 100 mL of MeOH for 24 h. Extraction with MeOH was repeated once more. The MeOH extracts obtained after the first and second maceration operations were combined and the MeOH was removed under vacuum. The yields of *O. bourgaei* and *O. trachytricha* extracts stored at +4 °C were 11.71% and 6.32% (*w*/*w*), respectively.

### 4.3. Chemical Composition Analysis

Chemical compositions of *O. bourgaei* and *O. trachytricha* were first analyzed qualitatively [48]. Subsequently, amounts of selected compounds in the extracts were determined chromatographically [49].

For total phenolic content, sample solution (0.25 mL) was mixed with diluted Folin-Ciocalteu reagent (1 mL, 1:9) and shaken vigorously. After 3 min, Na_2_CO_3_ solution (0.75 mL, 1%) was added and the sample absorbance was read at 760 nm after 2 h incubation at room temperature. Total phenolic content was expressed as equivalents of gallic acid.

For total flavonoid content, sample solution (1 mL) was mixed with the same volume of aluminium trichloride (2%) in methanol. Similarly, a blank was prepared by adding sample solution (1 mL) to methanol (1 mL) without AlCl_3_. The sample and blank absorbance were read at 415 nm after 10 min incubation at room temperature. Absorbance of the blank was subtracted from that of the sample. Total flavonoid content was expressed as equivalents of quercetin.

A 1260 Infinity liquid chromatography system (Agilent Technologies, Santa Clara, CA, USA) hyphenated to a 6420 Triple Quad mass spectrometer was used for quantitative analyses. Chromatographic separation was carried out on a Poroshell 120 EC-C18 (100 mm × 4.6 mm I.D., 2.7 μm) column. Three mobile phases were tested to obtain a complete resolution of all isomers and the highest sensitivity for all target compounds, namely: (i) 0.1% formic acid/methanol, (ii) 5 mM ammonium acetate/acetonitrile with 0.1% acetic acid and (iii) 10 mM ammonium formate with 0.1% formic acid/acetonitrile with 0.1% formic acid, respectively. The first mobile phase configuration (0.1% formic acid/methanol) was selected on the base of the better chromatographic resolution of isomeric compounds. On the other hand, the selected mobile phase configuration also provided higher sensitivity for many of the phenolic compounds. As a result, the mobile phase was made up from solvent A (0.1%, *v*/*v* formic acid solution) and solvent B (methanol). The gradient profile was set as follows: 0.00 min 2% B eluent, 3.00 min 2% B eluent, 6.00 min 25% B eluent, 10.00 min 50% B eluent, 14.00 min 95% B eluent, 17.00 min 95% B and 17.50 min 2% B eluent. The column temperature was maintained at 25 °C. The flow rate was 0.4 mL min^−1^ and the injection volume was 2.0 μL.

The tandem mass spectrometer was interfaced to the LC system via an ESI source. The electrospray source of the MS was operated in negative and positive multiple reaction monitoring (MRM) mode and the interface conditions were as follows: capillary voltage of −3.5 kV, gas temperature of 300 °C and gas flow of 11 L min^−1^. The nebulizer pressure was 40 psi.

### 4.4. Antioxidant and Enzyme Inhibitory Activities

Antioxidant [48,50,51,52,53] and enzyme inhibitory activities of *O. bourgaei* and *O. trachytricha* extracts were carried out following the methods given in the literature [28]. Total antioxidant activity of the samples was evaluated by phosphomolybdenum method. Sample solution (0.2 mL) was combined with 2 mL of reagent solution (0.6 M sulfuric acid, 28 mM sodium phosphate and 4 mM ammonium molybdate). The sample absorbance was read at 695 nm after 90 min incubation at 95 °C.

For 1,1-diphenyl-2-picrylhydrazyl (DPPH) radical scavenging activity, sample solution (1 mL) was added to a 4 mL of 0.004% methanol solution of DPPH. Sample absorbance was read at 517 nm after 30 min incubation at room temperature in dark.

For ABTS cation radical scavenging activity, briefly, ABTS^+^ radical cation was produced directly by reacting 7 mM ABTS solution with 2.45 mM potassium persulfate and allowing the mixture to stand for 12–16 h in dark at the room temperature. Prior to beginning the assay, ABTS solution was diluted with methanol to obtain an absorbance of 0.700 ± 0.02 at 734 nm. Sample solution (1 mL) was added to ABTS solution (2 mL) and mixed. Sample absorbance was read at 734 nm after 7 min incubation at room temperature.

For metal chelating activity on ferrous ions, briefly, sample solution (2 mL) was added to FeCl_2_ solution (0.05 mL, 2 mM). The reaction was initiated by the addition of 5 mM ferrozine (0.2 mL). Similarly, a blank was prepared by adding sample solution (2 mL) to FeCl_2_ solution (0.05 mL, 2 mM) and water (0.2 mL) without ferrozine. Then, the sample and blank absorbance were read at 562 nm after 10 min incubation at room temperature.

For cupric ion reducing activity (CUPRAC), sample solution (0.5 mL) was added to a premixed reaction mixture containing CuCl_2_ (1 mL, 10 mM), neocuproine (1 mL, 7.5 mM) and NH_4_Ac buffer (1 mL, 1 M, pH 7.0). Similarly, a blank was prepared by adding sample solution (0.5 mL) to a premixed reaction mixture (3 mL) without CuCl_2_. Then, the sample and blank absorbance were read at 450 nm after 30 min incubation at room temperature.

For ferric reducing antioxidant power (FRAP), sample solution (0.1 mL) was added to a premixed FRAP reagent (2 mL) containing acetate buffer (0.3 M, pH 3.6), 2,4,6-tris(2-pyridyl)- s-triazine (TPTZ) (10 mM) in 40 mM HCl and ferric chloride (20 mM) in a ratio of 10:1:1 (*v*/*v*/*v*). Then, the sample absorbance was read at 593 nm after 30 min incubation at room temperature.

Inhibitory activity on α-amylase was performed using Caraway-Somogyi iodine/potassium iodide (IKI) method. Sample solution (25 µL) was mixed with α-amylase solution (50 µL) in phosphate buffer (pH 6.9 with 6 mM sodium chloride) in a 96-well micro plate and incubated for 10 min at 37 °C. After pre-incubation, the reaction was initiated by the addition of starch solution (50 µL, 0.05%). Similarly, a blank was prepared by adding sample solution to all reaction reagents without enzyme solution (α-amylase). The reaction mixture was incubated 10 min at 37 °C. The reaction was then stopped with the addition of HCl (25 µL, 1 M). This was followed by the addition of iodine-potassium iodide solution (100 µL). The sample and blank absorbance were read at 630 nm. Absorbance of the blank was subtracted from that of the sample.

Tyrosinase inhibitory activity was measured using a modified dopachrome method with L- DOPA as substrate. Sample solution (25 µL) was mixed with tyrosinase solution (40 µL) and phosphate buffer (100 µL, pH 6.8) in a 96 -well microplate and incubated for 15 min at 25 °C. The reaction was then initiated with the addition of L-DOPA (40 µL). Similarly, a blank was prepared by adding sample solution to all reaction reagents without enzyme (tyrosinase) solution. The sample and blank absorbance were read at 492 nm after 10 min incubation at 25 °C.

Cholinesterase (ChE) inhibitory activity was measured using Ellman’s method. Sample solution (50 µL) was mixed with DTNB (125 µL) and AChE (or BChE) solutions (25 µL) in Tris-HCl buffer (pH 8.0) in a 96-well microplate and incubated for 15 min at 25 °C. The reaction was then initiated with the addition of acetylthiocholine iodide (ATCI) or butyrylthiocholine chloride (BTCl) (25 µL). Similarly, a blank was prepared by adding sample solution to all reaction reagents without enzyme solutions (AChE or BChE). The sample and blank absorbance were read at 405 nm after 10 min incubation at 25 °C. Absorbance of the blank was subtracted from that of the sample.

For α-glucosidase inhibitory activity, sample solution (50 µL) was mixed with glutathione (50 µL), α-glucosidase solution (50 µL) in phosphate buffer (pH 6.8) and PNPG (50 µL) in a 96-well microplate and incubated for 15 min at 37 °C. Similarly, a blank was prepared by adding sample solution to all reaction reagents without enzyme (α-glucosidase) solution. The reaction was then stopped with the addition of sodium carbonate (50 µL, 0.2 M). The sample and blank absorbance were read at 400 nm. Absorbance of the blank was subtracted from that of the sample.

The in vitro activities of the extracts were expressed as mg standard equivalent/g extract and compared with those of the standards, including trolox, ethylenediaminetetraacetic acid (disodium salt) (EDTA), galanthamine, kojic acid, and acarbose, used as positive controls.

### 4.5. In Silico Molecular Docking Analysis

The protein data bank (pdb) files of all the ligands (luteolin-7-glucoside, rosmarinic acid and apigenin-7-glucoside) were downloaded from PubChem (https://pubchem.ncbi.nlm.nih.gov) (accessed on 5 March 2021). In the Avogadro platform, the atom types and electrical charges of the ligands were optimized with MMFF94 force field using the steepest descent algorithm. The 3D structures of human pancreatic alpha-amylase (PDB ID: 1B2Y), human butyrylcholinesterase (PDB ID: 4BDS), recombinant human acetylcholinesterase (PDB ID: 4EY6), tyrosinase from *Bacillus megaterium* (PDB ID: 5I38) and human lysosomal acid-alpha-glucosidase (PDB ID: 5NN8) were downloaded from protein data bank (https://www.rcsb.org/) (accessed on 5 March 2021) and thereafter the crude protein structures were refined by removing redundant subunits, bound inhibitors and all other heteroatoms which are not functional in molecular docking simulations. The regions where the catalytic amino acids of the enzymes are located were determined by viewing the amino acid residues with which the co-crystallized ligand interacts in the Discovery Studio v16 software.

Molecular docking simulations between five enzymes and three ligands were undertaken using AutoDock 4.2.6 and the docking scores (binding free energies) of the ligands against pancreatic alpha-amylase, butyrylcholinesterase, recombinant human acetylcholinesterase, tyrosinase and alpha-glucosidase were subsequently calculated. In addition, for internal control purposes, enzymes were also redocked with their co-crystallized ligands and these binding affinity values obtained were used as positive controls for comparison [54,55,56]. For the preparation of the target and ligand molecules as well as the parameters prior to initiating the docking simulations using AutoDock 4.2.6, AutoDockTools-1.5.6 was used.

In this study, the grid box coordinates used in molecular docking simulations were adjusted to ensure that the tested three phytochemicals interact with the catalytic amino acid residues in the binding cavities of the enzymes in question.

Before molecular docking simulations, polar hydrogen atoms of the receptor and the ligand molecules were retained, while nonpolar hydrogens were merged and then, the Gasteiger charges of the ligands were calculated and the Kollmann charges were added for all the receptors with AutoDockTools-1.5.6. During the docking experiments, all the rotatable bonds of the ligands were set free and then the optimized protein (rigid) and ligand (flexible) structures were saved in PDBQT format. Grid box sizes were adjusted as: (a) 82 × 56 × 54 Å points for the acetylcholinesterase; (b) 70 × 62 × 54 Å points for butyrylcholinesterase; (c) 48 × 50 × 52 Å points for tyrosinase; (d) 40 × 40 × 40 Å points for alpha-amylase and, (e) 50 × 40 × 50 Å points for alpha-glucosidase, respectively. These grid box sizes were initially determined to cover the catalytic amino acid residues of all the enzymes studied.

In all docking simulations, 100 genetic algorithm (GA) runs using an initial population of 150 individuals, maximum number of 5,000,000 energy evaluations, and a maximum number of 27,000 generations were selected. The values of 0.02 and 0.8 were chosen as the default parameters for mutation and crossover rates, respectively. After 100 independent docking calculations, all the possible binding modes (conformations) of the ligands were clustered by the program and were ranked based on the most negative binding free energy (kcal/mol) of the ligand conformation. The best docking poses obtained using the AutoDock 4.2.6 between the ligand and receptor structures were analyzed with the BIOVIA Discovery Studio Visualizer v16.

### 4.6. Statistical Analysis

All in vitro tests were repeated three times to increase the scientific consistency of the results. The results are presented as mean value and standard deviation (mean ± SD). Student’s *t*-test with α = 0.05 (SPSS v. 22.0) was applied to detect the statistical similarities/differences between the data.

## 5. Conclusions

The fact that *O. trachytricha*, which was richer in most of the phytochemicals given in Table 2, is more active than *O. bourgaei* as an antioxidant, as expected, confirmed the view that the chemical composition has a determining effect on antioxidant activity. However, as evident from enzyme inhibition tests, *O. trachytricha* extract showed significantly higher activity only in BChE inhibition test compared to *O. bourgaei* extract. This suggests that specific components, rather than the overall extract composition, are responsible for enzyme inhibitory activity. When evaluated in combination with the results obtained from molecular docking analyses, it was found that the major components luteolin 7-glucoside and apigenin 7-glucoside showed the most important effect in enzyme inhibition activity and these two compounds can be recommended further as useful ligands for the inhibition of key enzymes that cause Alzheimer’s disease, diabetes and hyperpigmentation disorder. Hopefully, these compounds have been experimentally shown to cross the blood-brain barrier [57,58].

## Figures and Tables

**Table 1:** Analytical characteristics of selected phenolic compounds in the methanolic extracts from *O. bourgaei* and *O. trachytricha* ^x^.

Rt (min)	Compounds	Linear Equation	R^2^	LOD (μg/L)	LOQ (μg/L)
8.891	Gallic acid	y = 4.82x − 26.48	0.9988	1.46	4.88
10.818	Protocatechuic acid	y = 5.65x − 9.99	0.9990	1.17	3.88
11.224	3,4-Dihydroxyphenylacetic acid	y = 5.13x − 12.39	0.9990	1.35	4.51
11.369	(+)-Catechin	y = 1.45x + 1.95	0.9974	3.96	13.20
11.506	Pyrocatechol	y = 0.11x − 0.52	0.9916	9.62	32.08
11.802	Chlorogenic acid	y = 12.14x + 32.34	0.9995	0.55	1.82
12.412	2,5-Dihydroxybenzoic acid	y = 3.79x − 14.12	0.9980	2.12	7.08
12.439	4-Hydroxybenzoic acid	y = 7.62x + 22.79	0.9996	1.72	5.72
12.458	(−)-Epicatechin	y = 9.11x − 9.99	0.9971	1.85	6.18
12.841	Caffeic acid	y = 11.09x + 16.73	0.9997	3.15	10.50
12.843	Vanillic acid	y = 0.49x − 1.61	0.9968	2.56	8.54
12.963	Syringic acid	y = 0.74x − 1.54	0.9975	3.75	12.50
13.259	3-Hydroxybenzoic acid	y = 3.69x − 12.29	0.9991	1.86	6.20
13.397	Vanillin	y = 2.02x + 135.49	0.9926	15.23	50.77
13.589	Verbascoside	y = 8.59x − 28.05	0.9988	0.82	2.75
13.909	Taxifolin	y = 12.32x + 9.98	0.9993	1.82	6.05
13.992	Sinapic acid	y = 2.09x − 6.79	0.9974	2.64	8.78
14.022	p-Coumaric acid	y = 17.51x + 53.73	0.9997	1.93	6.44
14.120	Ferulic acid	y = 3.32x − 4.30	0.9992	1.43	4.76
14.266	Luteolin-7-glucoside	y = 45.25x + 156.48	0.9996	0.45	1.51
14.412	Hesperidin	y = 5.98x + 0.42	0.9993	1.73	5.77
14.506	Hyperoside	y = 16.32x − 1.26	0.9998	0.99	3.31
14.600	Rosmarinic acid	y = 9.82x − 17.98	0.9989	0.57	1.89
14.781	Apigenin-7-glucoside	y = 21.33x − 31.69	0.9983	0.41	1.35
15.031	2-Hydroxycinnamic acid	y = 16.72x − 26.94	0.9996	0.61	2.03
15.118	Pinoresinol	y = 0.80x − 2.69	0.9966	3.94	13.12
15.247	Eriodictyol	y = 14.24x − 0.50	0.9998	0.80	2.68
15.668	Quercetin	y = 14.68x − 18.25	0.9997	1.23	4.10
15.923	Luteolin	y = 8.96x + 26.80	0.9992	1.34	4.46
16.236	Kaempferol	y = 0.82x − 3.06	0.9959	3.30	10.99
16.382	Apigenin	y = 11.29x + 38.05	0.9987	0.96	3.20

^x^ Rt, retention time. LOD and LOQ: limit of detection and limit of quantification, respectively.

**Table 2:** Amounts of selected compounds in *Onosma* species ^x^.

Compound	*O. bourgaei* (µg/g)	*O. trachytricha* (µg/g)
Gallic acid	5.30 ± 0.16 ^a^	4.47 ± 0.25 ^a^
Protocatechuic acid	35.53 ± 0.57 ^a^	138.34 ± 2.44 ^b^
3,4-Dihydroxyphenylacetic acid	nd	11.40 ± 0.29
(+)-Catechin	nd	nd
Pyrocatechol	nd	nd
Chlorogenic acid	18.52 ± 0.39 ^a^	136.40 ± 10.35 ^b^
2,5-Dihydroxybenzoic acid	71.05 ± 0.61 ^a^	163.26 ± 0.93 ^b^
4-Hydroxybenzoic acid	297.18 ± 1.54 ^a^	392.63 ± 22.47 ^b^
(−)-Epicatechin	nd	nd
Caffeic acid	142.69 ± 4.28 ^a^	465.37 ± 16.12 ^b^
Vanillic acid	57.43 ± 2.39 ^a^	943.21 ± 14.90 ^b^
Syringic acid	13.76 ± 1.38 ^a^	32.83 ± 1.83 ^b^
3-Hydroxybenzoic acid	5.63 ± 0.36 ^a^	6.27 ± 0.06 ^a^
Vanillin	13.83 ± 0.96 ^a^	127.75 ± 3.12 ^b^
Verbascoside	1.89 ± 0.01	nd
Taxifolin	nd	nd
Sinapic acid	18.72 ± 0.41 ^a^	39.65 ± 2.26 ^b^
*p*-Coumaric acid	58.46 ± 0.39 ^a^	119.23 ± 5.30 ^b^
Ferulic acid	112.78 ± 0.94 ^a^	559.18 ± 47.28 ^b^
Luteolin 7-glucoside	23,908.22 ± 922.89 ^a^	22,326.01 ± 216.87 ^a^
Hesperidin	8.63 ± 0.01 ^a^	79.04 ± 2.02 ^b^
Hyperoside	19.47 ± 0.10 ^a^	120.51 ± 4.32 ^b^
Rosmarinic acid	10,013.76 ± 175.93 ^a^	24,837.51 ± 1069.14 ^b^
Apigenin 7-glucoside	21,689.17 ± 215.93 ^b^	17,949.03 ± 468.50 ^a^
2-Hydroxycinnamic acid	nd	nd
Pinoresinol	1224.04 ± 1.94 ^b^	762.64 ± 36.65 ^a^
Eriodictyol	2.47 ± 0.08	nd
Quercetin	2.75 ± 0.06 ^a^	3.21 ± 0.23 ^a^
Luteolin	1890.26 ± 85.62	nd
Kaempferol	nd	3.97 ± 0.18
Apigenin	211.32 ± 1.04 ^a^	280.06 ± 7.68 ^b^

^x^ Data with different superscripts (a, b) within the same row were different from each other. nd—not detected.

**Table 3:** Binding affinity (binding free energy) and calculated inhibition constant values of luteolin 7-glucoside, apigenin 7-glucoside and rosmarinic acid against enzymes in molecular docking simulations.

Enzyme	Ligand	Binding Affinity (ΔG°; kcal/mol)	Inhibition Constant (mM)
AChE	Galantamine (inhibitor)	−7.25	0.0048
	Luteolin 7-glucoside	−8.92	0.0003
	Apigenin 7-glucoside	−9.26	0.0001
	Rosmarinic acid	−6.72	0.0110
BChE	Tacrine (inhibitor)	−6.93	0.0082
	Luteolin 7-glucoside	−10.87	0.00001
	Apigenin 7-glucoside	−9.86	0.00006
	Rosmarinic acid	−7.91	0.0015
Tyrosinase	Kojic acid (inhibitor)	−5.64	0.0728
	Luteolin 7-glucoside	−5.83	0.0536
	Apigenin 7-glucoside	−5.41	0.1081
	Rosmarinic acid	−4.58	0.4383
α-Amylase	Acarbose (inhibitor)	−10.4	0.00002
	Luteolin 7-glucoside	−8.19	0.0009
	Apigenin 7-glucoside	−7.64	0.0025
	Rosmarinic acid	−6.17	0.0301
	Acarbose (inhibitor)	−9.53	0.0001
	Luteolin 7-glucoside	−9.22	0.0001
α-Glucosidase	Apigenin 7-glucoside	−9.09	0.0002
	Rosmarinic acid	−5.26	0.139

## Data Availability

The data presented in this study are available on request from the corresponding author.

## Sample Availability

Samples of the compounds are not available from the author.
